# The impact of cigarette smoking in predicting stroke using CHADS_2_ and CHA_2_DS_2_-VASc schemas

**DOI:** 10.1007/s10072-020-04455-w

**Published:** 2020-06-22

**Authors:** Ming-Liang Zuo, Chun-Mei Li, Yan Deng, Sanjib Bhattacharyya, Ping Shuai, Hung-Fat Tse, Chung-Wah Siu, Li-Xue Yin

**Affiliations:** 1 Department of Cardiovascular Ultrasound and Non-invasive Cardiology, Health Management Center, Sichuan Provincial People’s Hospital, Affiliated Hospital of University of Electronic Science and Technology, 32# W. Sec 2, 1st Ring Rd, Chengdu, 610072 China; 2 College of Pharmaceutical Sciences, Southwest University, Beibei, Chongqing, 400715 China; 3 Cardiology Division, Department of Medicine, Queen Mary Hospital, The University of Hong Kong, Room 1928, Block K, 102 Pokfulam Road, Hong Kong SAR, 999077 China

**Keywords:** Ischemic stroke, Cigarette smoking, Risk prediction, CHADS_2_, CHA_2_DS_2_-VASc

## Abstract

**Objective:**

To determine the impact of smoking status in the prediction of stroke using CHADS_2_ and CHA_2_DS_2_-VASc schemes.

**Methods:**

Five hundred twenty-eight consecutive patients with arrhythmic symptoms and without any documented arrhythmia from Queen Mary Hospital, Hong Kong, were followed up to determine the incidence of ischemic stroke, new-onset atrial fibrillation (AF), or all-cause mortality. Smoking status was classified into nonsmokers and smokers. The pairwise comparisons of C-statistics for outcomes were performed.

**Results:**

During a median follow-up period of 6.2 years, 65 (12.3%) individuals developed ischemic stroke. Smokers experienced higher annual incidence of stroke, a new-onset AF, and all-cause death compare to nonsmokers, with corresponding hazard ratio (HR) of stroke, AF, and all-cause death being 2.51 (95% confidence intervals, CI 1.36als, CIse death bein 1.15a3.24), and 1.95 (95% CI 1.161.95 (95% CIath being 2.51 (95% confidence corr_2_ and CHA_2_DS_2_-VASc for stroke were 0.60 (95% CI 0.51 for st*p* = 0.09) and 0.59 (95% CI 0.50 (95%, *p* = 0.15) respectively, whereas the C-statistics of CHADS_2_ and CHA_2_DS_2_-VASc were 0.66 (95% CI 0.61 were 0*p* = 0.005), 0.75 (95% CI 0.7 CI 0.7*p* < 0.0001), respectively among nonsmokers. After incorporating smoking, both the CHADS_2_-smoking and CHA_2_DS_2_-VASc-smoking achieved better C-statistics for new-onset ischemic stroke prediction superior to baseline score systems in male groups.

**Conclusion:**

Cigarette smoking status has impact on stroke stratification using CHADS_2_ and CHA_2_DS_2_-VASc scheme. The discrimination of the CHADS_2_ and CHA_2_DS_2_-VASc scheme for stroke can be significantly improved if smoking status is additionally considered.

## Introduction

Stroke that often shows up unnoticed in our life remains a major healthcare problem. Howard et al. forecasted that the number of stroke events will dramatically increase (more than double) from 2010 to 2050, and the increased burden of care of stroke patients on an already stressed healthcare system could be overwhelming [1]. Not only will this burden fall on physicians and hospitals, rather enhance the demand for rehabilitation services and increased nursing home. Therefore, early identification of individual with risk might enable a closer surveillance for the susceptibility of stroke incident and therefore prompt initiation of oral anti-platelet or anticoagulation treatment for stroke prevention.

Generally, current stroke risk stratification schemas such as CHADS_2_, CHA_2_DS_2_-VASc, and NICE are validated stratification tools to estimate the risk factor of stroke occurrence used as guideline for oral anticoagulation therapy among non-valvular atrial fibrillation (NVAF) patients [2, 3], though Trousseau score recently appeared for differentiating cancer-associated stroke in patients with cancer [4]. Compared to the CHADS_2_ score, the CHA_2_DS_2_-VASc score includes three additional risk factors: female, age 64–75, and vascular disease for ischemic stroke, whereas cigarette smoking status is not considered during risk assessment in all those validation cohorts mentioned above. Cigarette smoking is a well-known risk factor for ischemic stroke and associated with an approximate doubling of risk for ischemic stroke after adjustment for other risk factors [5, 6]. Smoking status relates to atherosclerosis, vascular damage (e.g., endothelial dysfunction), AF incidence (e.g., increased atrial fibrosis), and the incidence of mild cognitive impairment as well [7, 8].

In recent studies, CHADS_2_ and CHA_2_DS_2_-VASc scores have been reported with similar (modest) predictive capacity for stroke in general population and non-AF patient populations including the risk of death after stroke, new-onset AF, and stroke in unselected patients [9–11]. However, little is known about the impact of smoking on the incidence of stroke in patients with arrhythmic symptoms but no AF recorded. In the present study, we determined the impact of smoking status for the prediction of stroke using CHADS_2_ and CHA_2_DS_2_-VASc schemes.

## Patients and methods

### Design and sampling

The cohort study was established in Hong Kong and approved by the local ethics committee, and individual informed consent was obtained from all subjects. The study design has been reported in detail elsewhere [12]. In brief, 743 consecutive patients were referred to the cardiac clinic of Queen Mary Hospital, Hong Kong for assessment of palpitation, dizziness, and/or syncope.

### Data collection and clinical evaluation

For each patient, demographics at baseline, detailed medical history, medication use, and cardiovascular diseases were recorded. Thorough clinical examination, standard 12-lead electrocardiogram, Holter, and conventional echocardiographic examination were performed. Left ventricular ejection fraction was measured by echocardiography using a biplane modified Simpson’s method with the GE Vivid 7 (GE Healthcare, Milwaukee, Wisconsin). Blood samples were obtained by venipuncture on the same day of the procedure after a 12-h overnight fast and drew into standardized tubes that were delivered to the laboratory within a few minutes. Fasting blood glucose, total cholesterol, low-density lipoprotein cholesterol (LDL-C), high-density lipoprotein cholesterol (HDL-C), triglycerides, and renal function tests were performed using standard laboratory methods. Diabetes was considered present if fasting blood glucose 126 mg/dl or low-density lipoprotein cholesterol (LDL-C), high-density lipoprotein cholesterol (HDL-C), triglycerides, and renal function tests were performed using standard laboratory methods. Diabetes was considered present if the fasting blood glucose was higher than 126 mg/dl or the subject was taking antidiabetic medication. Hypertension was defined as systolic and diastolic blood pressures are higher than 140 mmHg and 90 mmHg, respectively, or if the subject was taking antihypertensive medication. Heart failure and history of stroke/TIA in medical record, and vascular diseases including myocardial infarction, peripheral artery disease, and complex aortic plaque were evaluated [3].

### Smoking status

Smoking status was classified as follows: Nonsmokers were defined as former smokers and individuals who never smoked. Current-smokers were defined as those who have been smoking at least one cigarette per day [13]. We also grouped current smokers according to their cigarettes consumption, as follows: < 10, 10–19, and ≥ 20 cigarettes/day.

### Risk score calculation

The CHADS_2_ score was calculated as the following: 1 point was assigned to chronic heart failure, hypertension (HT), age over 75 years, and diabetes mellitus (DM), respectively; 2 points was assigned to the history of stroke. The CHA_2_DS_2_-VASc score is a modification of the CHADS_2_ score by adding 1 point each for three additional risk factors: vascular disease (V), age of 65 to 74 years (A), and gender of female (as a sex category). The CHADS_2_-S or CHA_2_DS_2_-VASc-S score adds smoking (S) to the previous scores. The maximum CHADS_2,_ CHA_2_DS_2_-VASc, CHADS_2_-S, and CHA_2_DS_2_-VASc-S were 6, 9, 7, and 10 respectively.

### Follow-up and end point

Patients were followed up to determine the incidence of the primary end point of ischemic stroke, new-onset AF, or all-cause mortality. Information on the end points was collected from hospital databases and responses to questionnaires by patients themselves or their family members. The new occurrence of clinical AF was defined as the presence of AF documented by resting 12-lead ECGs. Ischemic stroke was defined as a neurological deficit of sudden onset that persisted for more than 24 h in the absence of intracerebral and subarachnoid hemorrhage, and that could not be explained by other causes (trauma, infection, and vasculitis). Stroke was confirmed by computerized axial tomography or magnetic resonance imaging of the brain. Death events were determined by a query of computerized social security death records and the medical records.

### Statistical analyses

The baseline information is summarized as mean ± standard deviation for continuous variables and as frequencies for discrete variables. Comparisons between the smoker and nonsmoker groups were performed using Student us*t* test. Categorical variables were summarized as percentages and compared with the chi-square or Fisherrized as perc. Hazard ratios (HRs) and their 95% confidence intervals (CIs) were computed by means of time-dependent Cox regression models. To predict stroke or AF and death, the discriminatory power toward CHADS_2_ and CHA_2_DS_2_-VASc was quantified by determining the area under the receiver operator characteristic (ROC). The pairwise comparisons of ROC curves for each outcome were performed. Data were analyzed with SPSS 15.0 (SPSS, Inc. Chicago, IL) and MedCalc Statistical Software11.4 (MedCalc Software bvba, Ostend, Belgium). A *p* value < 0.05 was considered nominally significant. Ideal prediction yields a C-statistics of 1.00, whereas a value of < 0.5 reflects that prediction is no better than chance.

## Results

### Baseline characteristics and risk scores by smoking

A total of 743 consecutive patients were admitted for the study, and 215 were excluded due to any documented high-grade atrioventricular block and/or sustained cardiac arrhythmias including AF during resting ECG and 24-h ECGs, the presence of any implantable pacemaker or cardioverter defibrillators, hyperthyroidism, major valvular heart diseases, or incomplete clinical or follow-up data. As a result, a total of 528 Chinese and other Asian participants, including 145 smokers and 383 nonsmokers, were registered for clinical trial and follow-up studies. The participants have an average age of 68.5 years and 46.2% were male; 45.3% had hypertension; 17.1% had diabetes mellitus; 18.5% had coronary artery disease (CAD).

The mean age of the smokers was 67 years. The percentage of smokers in male group was higher than in female group. Among the smoker’s group, 48.3% of them received antiplatelet administration. The nonsmokers had an average age of 66 years and 32.0% of them had antiplatelet therapy. The prevalence of CAD is higher among smokers than nonsmokers. There is a higher portion of smokers than nonsmokers who have CHADS_2_ score equal or more than 2. There is no difference between the CHA_2_DS_2_-VASc scores of smokers and nonsmokers across different strata (Table 1).

**Table 1:** Baseline characteristics and risk scores by cigarette smoking

	Total population (*n* = 528)	Nonsmokers (*n* = 383)	Smokers (*n* = 145)	*p*
Mean age (SD, years)	66.7 ± 10.2	65.7 ± 9.9	68.9 ± 10.4	0.004*
Age ≥ 75, *n* (%)	138 (26.1)	84 (21.9)	54 (37.3)	0.001*
Age 65–70, *n* (%)	159 (30.1)	121 (31.6)	38 (26.3)	0.005*
Male, *n* (%)	230 (43.7)	113 (29.6)	117 (80.5)	0.000*
CHF, *n* (%)	--	--	--	
HT, *n* (%)	239 (45.3)	171 (44.8)	68 (46.6)	0.74
DM, *n* (%)	90 (17.1)	59 (15.5)	31 (21.2)	0.16
CAD, *n* (%)	92 (17.5)	57 (14.8)	35 (24.6)	0.02*
Pre stroke /TIA, *n* (%)	---	---	---	
Echo				
LAD (mm)	3.8 ± 0.8	3.8 ± 0.8	3.9 ± 0.8	0.38
LVEF (%)	65.8 ± 8.1	66.1 ± 7.8	64.8 ± 8.6	0.13
Anyantiplatelet therapy, *n* (%)	195 (36.9)	122 (32.0)	73 (48.3)	0.001*
CHADS_2_ score				0.03*
0	215 (40.6)	162 (42.2)	53 (36.4)	0.49
1	180 (34.1)	138 (36.1)	42 (28.8)	0.32
≥ 2	133 (25.1)	83 (21.6)	50 (34.7)	0.03
CHA_2_DS_2_-VASc score				
0	48 (9.1)	30 (7.7)	18 (12.7)	0.19
1	142 (26.9)	107 (28.1)	34 (23.7)	0.49
≥ 2	338 (62)	246 (69.2)	93 (63.6)	0.94

CHF indicates congestive heart failure; *HT*, hypertension; *DM*, diabetes mellitus; *CAD*, coronary artery disease; *TIA*, transient ischemic attack; *LAD*, left atrial dimension; *LVEF*, left ventricular ejection fraction; *CHADS2*, *C* congestive heart failure; *H*, hypertension; *A*, age >75; *D*, diabetes mellitus and *S*, prior stroke or transient ischemic attack; *CHA2DS2-VASc, C*, congetive heart failure; *H*, hypertension, *A2*, age 65 to 74 years and age ≥ 75 years; *D*, diabetes mellitus, and *S*, prior stroke or transient ischemic attack; *VA*, vascular disease; *Sc*, sex category. *p < 0.05

However, based on the CHA_2_DS_2_-VASc scores, the proportions of cigarette smokers with CHA_2_DS_2_-VASc score of 0 and 1 were heterogeneously distributed (*p* = 0.048) with 38% smokers in the score of 0 and with 24% smokers in the score of 1. The proportions of cigarette smokers with CHA_2_DS_2_-VASc score of 3 and ≥ 4 were also heterogeneously distributed (*p* = 0.040) with 38% smokers in the score of 3 and with 19% smokers in the score of ≥ 4. Whereas, there was no significant heterogeneity of cigarette smoking in CHADS_2_ score of 0 and 1, and 1 and ≥ 2 in this population (*p* = 0.77, 0.058 respectively).

### Comparison of hazard ratios by smoking

During the median follow-up period of 6.2 years, 65 (12.3%) developed ischemic stroke, 89 (16.8%) were newly diagnosed with AF, and 89 (16.8%) died in the study groups.

Table 2 shows the annual events rates in this population and hazard ratios for outcomes by smoking status. The risk of the endpoints was significantly higher among smokers during follow-up, with corresponding HR for stroke, new-onset AF, and death being 2.51 (95% CI 1.36% CIt, *p* = 0.003), 1.93 (95% CI 1.153), 1.9*p* = 0.01), and 1.95 (95% CI 1.16 1.95 (*p* = 0.01), respectively.

**Table 2:** Events rates by smoking status and hazard ratios for outcomes

	Event rate (%/year)	*p* value		HR (95% CI)		*p* value
	Nonsmokers	Smokers				
Ischemic stroke	0.042	0.069	0.002	2.51 (1.36–4.64)		0.003*
New-onset AF	0.059	0.112	0.01	1.93 (1.15–3.24)		0.01*
Death	0.065	0.113	0.02	1.95 (1.16–2.27)		0.01*

*Log-rank *p* value for nonsmokers versus smokers, *CI* confidence interval

### Comparison of annual event rates by risk scores and smoking

Higher incidence of annual risk of stroke was observed in CHA_2_DS_2_-VASc score of 0 than that of 1 (0.85 vs. 0.57, *p* = 0.048), although all patients with higher CHADS_2_ or CHA_2_DS_2_-VASc scores were more likely to suffer from ischemic stroke.

**Fig. 1:**
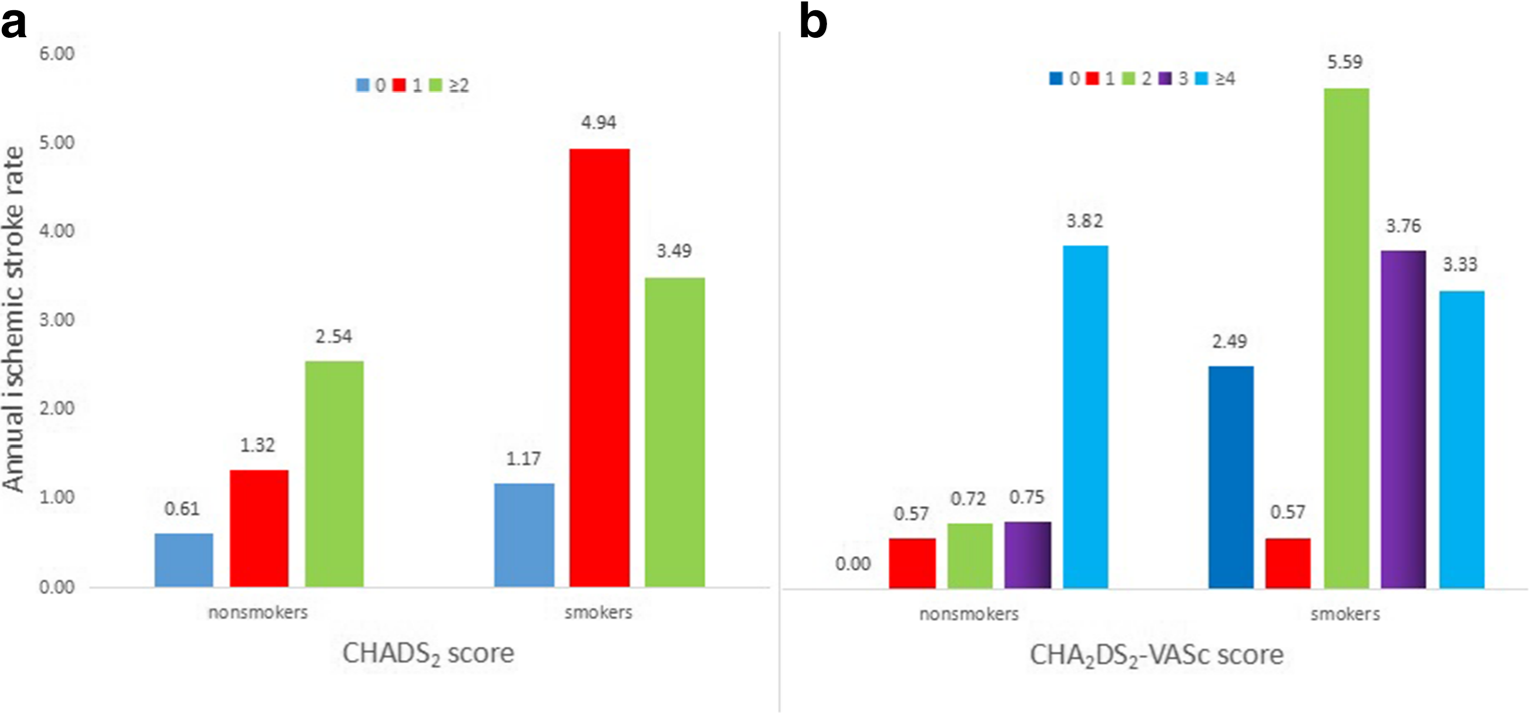
Annual event rates according to risk stratification in nonsmokers and smokers. **a** Cumulative event rate of patients with or without smoking by CHADS_2_ score. **b** Cumulative event rate of patients with or without smoking by CHA_2_DS_2_-VASc score

Figure 1 shows annual event rates according to different risk stratification in nonsmokers and smokers. There was significantly different risk trend of ischemic stroke between the two groups. Nonsmoker group with higher risk score experienced higher cumulative event rate stratified by either CHADS_2_ or CHA_2_DS_2_-VASc score. Moreover, no ischemic stroke events were recorded with CHA_2_DS_2_-VASc score of 0. However, smoker group with lower risk score suffered from higher risk trend of ischemic stroke during follow-up.

### The impact of smoking on stroke prediction by comparison of ROC

**Fig. 2:**
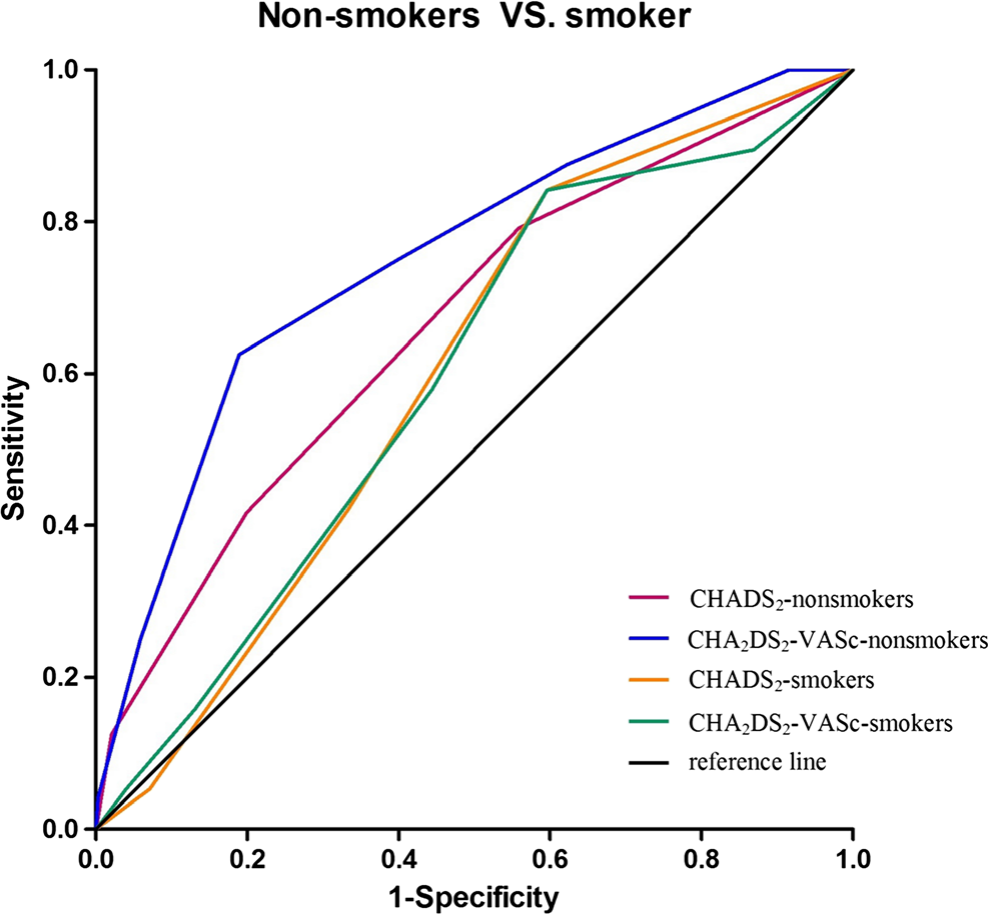
Receive-operating characteristic (ROC) curves for the performance of CHADS_2_ and CHA_2_DS_2_-VASc scores in predicting stroke according to cigarette smoking

We further performed receiver-operating characteristic analysis to determine the prediction for ischemic stroke, new-onset AF, and death using CHADS_2_ and CHA_2_DS_2_-VASc among nonsmokers and smokers. Table 3 presents the comparison of the two scoring systems for ischemic stroke, new-onset AF, and death. Among smokers, predictivity performance of the two schemes for ischemic stroke and death was poorer than that among nonsmokers. The area under the curve of CHADS_2_ and CHA_2_DS_2_-VASc for ischemic stroke among smokers were 0.60 (95% CI 0.5–0.69, *p* = 0.09) and 0.59 (95% CI 0.50–0.68, *p* = 0.15), respectively. Whereas, the area under the curve of CHADS_2_ and CHA_2_DS_2_-VASc for ischemic stroke among nonsmokers were 0.66 (95% CI 0.61Ifor is*p* = 0.005), 0.75 (95% CI 0.75), 0.7*p*< 0.0001), respectively. Figure 2 shows that nonsmokers had a higher area under the curve of CHADS_2_ and CHA_2_DS_2_-VASc for stroke compared to smokers subjects. Moreover, CHA_2_DS_2_-VASc score had also better performance than CHADS_2_ among nonsmokers.

**Table 3:** Comparison of ROC by smoking

Events	Parameter	C-statistics (95% CI)			
		Nonsmokers	*p*	Smokers	*p*
Stroke	CHADS_2_	0.66 (0.61–0.71)	0.005*	0.60 (0.51–0.69)	0.09
	CHA_2_DS_2_-VASc	0.75 (0.70–0.80)	0.0001*	0.59 (0.50–0.68)	0.15
AF	CHADS_2_	0.65 (0.59–0.70)	0.0003*	0.65 (0.55–0.73)	0.01*
	CHA_2_DS_2_-VASc	0.70 (0.65–0.75)	< 0.0001*	0.68 (0.59–0.76)	0.0016*
Death	CHADS_2_	0.61 (0.55–0.66)	0.02*	0.60 (0.51–0.69)	0.06
	CHA_2_DS_2_-VASc	0.70 (0.65–0.75)	< 0.0001*	0.60 (0.51–0.69)	0.08

*ROC* receiver operating characteristic curve; *p < 0.05

**Fig. 3:**
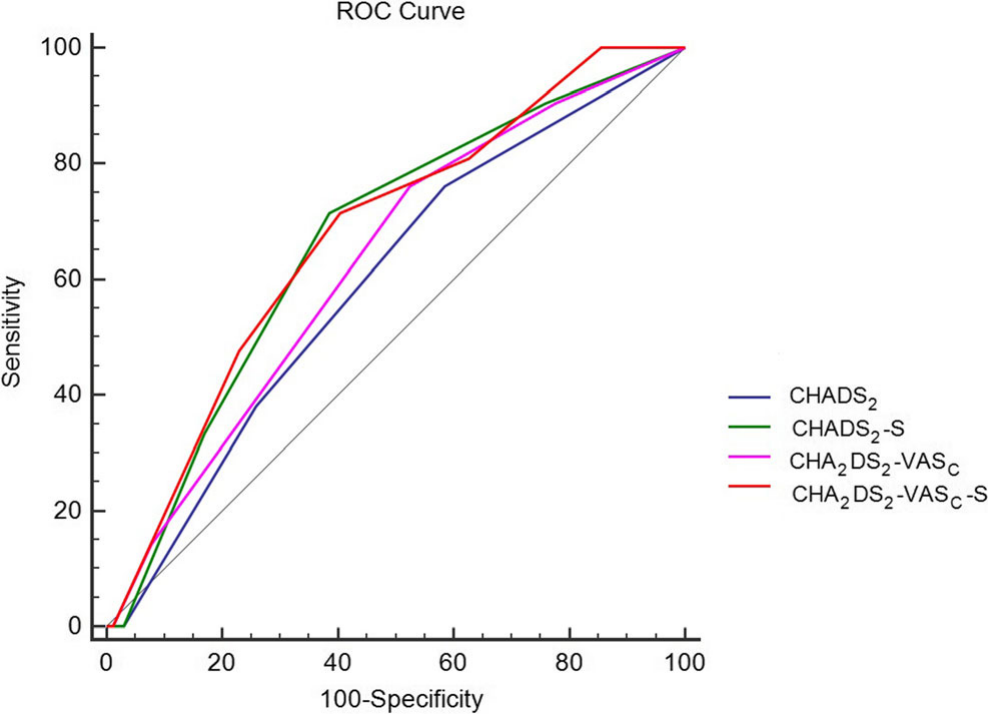
Receiver-operating characteristic (ROC) curves of CHADS_2_, CHA_2_DS_2_-VASc, and combined smoking scores for prediction of new-onset ischemic stroke

To account for the potential effect of cigarette smoking for predicting stroke incidence using CHADS_2_ and CHA_2_DS_2_-VASc schemas, we excluded female who seldom smoked. After incorporating smoking, CHADS_2_-S was better predictor for ischemic stroke compared to baseline CHADS_2_ (*p* = 0.006) in male groups. Similarly, CHA_2_DS_2_-VASc-S achieved better C-Statistic compared to baseline CHA_2_DS_2_-VASc score (*p* = 0.01, Fig 3) in male groups. The C-statistics with 95% confidence interval (95% CI) for that respective scores were CHADS_2_ 0.59 (95% CI 0.522–0.667, *p* = 0.10); CHADS_2_-S 0.67 (95% CI 0.596–0.736, *p* = 0.004); CHA_2_DS_2_-VASc 0.63 (95% CI 0.556–0.699, *p* = 0.03); and CHA_2_DS_2_-VASc-S 0.68 (95% CI 0.605–0.744, *p* = 0.002) in male groups.

## Discussion

In this study, we have demonstrated that cigarette smoking has impact on stroke risk stratification using CHADS_2_ and CHA_2_DS_2_-VASc schemes in patients with arrhythmic symptoms without documented AF. The relatively poorer performance of the risk scores was shown in smokers. The area under the ROC curve for CHA_2_DS_2_-VASc scores was significantly higher in nonsmokers than in smokers (0.75 vs. 0.59, *p*<0.05). After incorporating smoking, risk factor for ischemic stroke, to both the CHADS_2_-S and CHA_2_DS_2_-VASc-S score, we can get superior estimates in the risk prediction of ischemic stroke in male groups. Our results suggest that cigarette smoking status should be additionally considered when estimating the risk of ischemic stroke.

To our knowledge, this is the important study for the prediction of ischemic stroke among patients with arrhythmic symptoms without documented AF on presentation stratified by cigarette smoking. Previous studies have addressed the cigarette smoking as an independent risk factor for initial and recurrent ischemic stroke [14–16]. In addition, spousal smoking poses important stroke risks for never-smokers and former smokers (HR 1.42, 1.72, respectively) [17]. In the present study, HR for ischemic stroke among smoking patients is compared with non-smoking counterparts ranged from 1.36 to 4.64 (HR = 2.51, *p* = 0.003), which reaffirms that patients with cigarette smoking represent a “high-risk” population. Indeed, smoking continued to be a strong independent predictor for stroke occurrence [18], major adverse cardiac, and cerebrovascular events (MACCE) (OR = 2.34, 95% CI 1.49–3.68) after adjustment for clinical and angiographic variables in patients treated with drug-eluting stents [19]. By contrast, nonsmoking was a significant prognostic factor of favorable outcomes after ischemic stroke, and long-time smoking had a negative effect on stroke severity [20, 21].

On the other hand, lower score did not correspond to lower annual stroke rate when stratified by CHA_2_DS_2_-VASc score among all patients (0.85 in CHA_2_DS_2_-VASc score of 0, 0.57 in CHA_2_DS_2_-VASc score of 1) [12]. By contrast, we observed that the risk of stroke during follow-up increased with CHADS_2_ score, as it was double for patients with CHADS_2_ score of 1 compared with score of 0. This may be partially due to the heterogeneity of cigarette smoking, with higher proportion of cigarette smoking in CHA_2_DS_2_-VASc score of 0 compared with those in the score of 1. There was no significant heterogeneity of cigarette smoking in CHADS_2_ score of 0 and 1 in this population (*p* = 0.77). As a confounding factor, the heterogeneity of smoking maybe had some effect on the prediction performance of CHA_2_DS_2_-VASc schema. Indeed, during the subgroup analysis of smokers was excluded whereas nonsmokers with higher risk score experienced higher cumulative event rate and no ischemic stroke events were recorded with CHA_2_DS_2_-VASc score of 0 during follow-up. On the contrary, smokers with lower risk score suffered from higher ischemic stroke.

Since smokers had a higher risk of stroke events rate, how to improve risk stratification is an important issue. In the present study, both the C-statistics of CHADS_2_ and CHA_2_DS_2_-VASc in smokers were poorer than that in nonsmoking population. Moreover, CHA_2_DS_2_-VASc score performed better than CHADS_2_ among nonsmokers in predicting ischemic stroke assessed by the C-statistics, which suggested that cigarette smoking has impact on stroke risk stratification.

Existing schemes to predict ischemic stroke do not include cigarette smoking as an independent predictor for stroke events. Even it has been underrepresented in clinical trials and entirely absent from the completed double-blind trial of factors associated with ischemic stroke [22, 23], indeed, revised schemes must be comprehensive enough to incorporate all the independent variables that contribute importantly to the risk factors of stroke. Recently, efforts to improve risk stratification have been made. For example, the CHA_2_DS_2_-VASc-HS or CHA_2_DS_2_-VASc-HSF score, which includes hyperlipidemia (HL) and smoking (S) or family history(F), is found to be the best score scheme to predict CAD severity in comparison to ROC curves, such as a score > 2 may predict CAD severity [24, 25]. Therefore, further investigation is needed to develop more accurate stroke risk stratification scheme by incorporating cigarette smoking status to CHADS_2_ or CHA_2_DS_2_-VASc score schema.

In the present study, when additional smoking status was considered, we found that CHA_2_DS_2_-VASc score offered excellent predictability for stroke in nonsmokers and limited predictability in smokers (C-statistics 0.75 vs. 0.59, respectively). Additionally, real low risk of stroke (i.e., those entirely free of stroke events at 6.2 ± 1.3 years) can be identified by CHA_2_DS_2_-VASc score in nonsmokers. Our novel data suggests that incorporating smoking as a risk factor for ischemic stroke, to both the CHADS_2_ and CHA_2_DS_2_-VASc score, resulted in superior estimates for the risk prediction of ischemic stroke in male groups.

## Conclusion

In this population of patients that were referred for assessment of palpitation, dizziness, and/or syncope, smokers experienced higher hazard ratio (HR) of stroke, AF, and all-cause death. The discrimination of the CHADS_2_ and CHA_2_DS_2_-VASc scheme for stroke can be significantly improved when smoking status was additionally considered. Future studies are warranted to determine the smoking-specific risk stratification.

### Strengths and limitations

The present study was comprised of a single-center design with a relatively small sample size, and therefore the results might differ if a larger population were used. The study was based on patients who were admitted for assessment of palpitation, dizziness, and/or syncope, which may be observer bias. Therefore, the results need to be reaffirmed in a more representative large patient population.
